# Morphometric analysis of retroperitoneal organs in individuals with unilateral congenital renal agenesis and renal hypoplasia

**DOI:** 10.12669/pjms.42.1.13016

**Published:** 2026-01

**Authors:** Tayfun Aygun, Nurullah Yucel, Meral Mese, Ridvan Dizman, Tamer Baysal

**Affiliations:** 1 Tayfun Aygun Assistant Professor, Department of Anatomy, Faculty of Medicine, Giresun University, Giresun, Turkey; 2 Nurullah Yucel Assistant Professor, Department of Anatomy, Hamidiye International Faculty of Medicine, Health Sciences University, Istanbul, Turkiye; 3 Meral Mese Associate Professor, Nephrology Clinic, Department of Internal Medicine, Istanbul Dr. Lutfi Kirdar City Hospital, Health Sciences University, Istanbul, Turkiye; 4 Ridvan Dizman Research Assistant, Department of Radiology, Istanbul Dr. Lutfi Kirdar City Hospital, Health Sciences University, Istanbul, Turkiye; 5 Tamer Baysal Professor, Department of Radiology, Istanbul Dr. Lutfi Kirdar City Hospital, Health Sciences University, Istanbul, Turkiye

**Keywords:** Cakut, Congenital abnormalities, Kidney size, Renal agenesis, Renal hypoplasia

## Abstract

**Objective:**

Our aim was to determine the changing anatomy of the retroperitoneal organs in the Unilateral Congenital Renal Agenesis (UCRA) and Renal Hypoplasia (RH) groups and to investigate the presence of morphological biomarkers by evaluating the functional capacity of the kidney in the patient groups.

**Methodology:**

The study was conducted retrospectively at Istanbul Dr. Lütfi Kırdar City Hospital, in collaboration with the Departments of Radiology, Nephrology and Anatomy. CT images of individuals with UCRA and RH followed in the nephrology clinic were retrospectively reviewed and anatomical-topographic evaluations were performed. Biomarkers indicating renal function were obtained from laboratory test results. Statistical significance was accepted as p<0.05

**Results:**

The study included 18 individuals in the agenesis group, 30 in the hypoplasia group, and 25 in the control group. Compensatory hypertrophy was significantly observed in all renal morphometric measurements in the healthy side kidney. It was observed that the estimated glomerular filtration rate (eGFR) decreased from the control group to the agenesis group. The anteroposterior diameter of the inferior vena cava was narrower in individuals with CAKUT (n=48) than in controls (n=25) (CAKUT: 14.50±3.83, Control 17.10±3.80, p=0.007). Cortex thickness was positively correlated with eGFR and negatively correlated with creatinine in both the UCRA group and the HP group. The cortex thickness/mediolateral diameter ratio was negatively correlated with urea, uric acid, creatinine and positively correlated with eGFR.

**Conclusions:**

A narrowing of the VCI-AP diameter was notable in patients with CAKUT. Cortical thickness was considered important in the assessment of renal function, and the ratio of cortical thickness to mediolateral diameter (Cx/ML) could be used as a potential reference.

## INTRODUCTION

The formation of the kidneys begins in the 3rd week and continues until the 36th week. These structures, positioned from cranial to caudal as pronephros, mesonephros and metanephros, play a role in the filtration process of embryonic blood.^1^ Kidney development is sensitive to environmental risk factors throughout pregnancy.^2^ The fusion of the metanephric blastema with the ureteric bud activates kidney development.^3^ While nephron structures form from the metanephros, the collecting duct system develops from the ureteric bud. In normal development, the kidney weight and volume at birth are only approximately 10% of the mature kidney.^4^

Congenital anomalies of the kidney and urinary tract (CAKUT) refer to a wide range of structural abnormalities resulting from a defect in the embryonic development of the kidney and urinary tract.^5^ They cover 17.5-30% of all congenital malformations and are among the most common organ malformations.^6^,^7^ CAKUT has an important role in chronic renal failure and certain abnormalities have been reported to cause cardiovascular diseases and hypertension in adult life.^8^ Updated in February 2024, EUROCAT (European survey of congenital anomalies) defined CAKUT in 8 subgroups by adding three new subgroups and added unilateral renal agenesis.^9^ Unilateral congenital renal agenesis (UCRA) and renal hypoplasia (RH) are considered among the numerical and size anomalies of CAKUT.^10^

**Fig.1:**
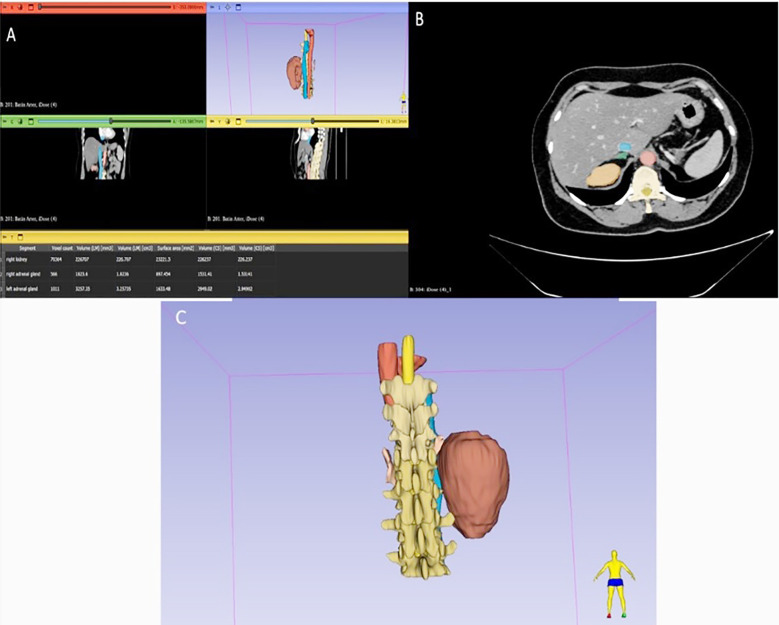
The Total Segmentator module of the 3D Slicer program was used to obtain renal volumes. A: All abdominal structures were obtained using automatic segmentation, and statistical data for the necessary structures were obtained from the “segment statistics” tab. B: In axial sections, blue represents the IVC, red represents the abdominal aorta, green represents the adrenal gland, and orange represents the renal parenchyma. C: The posterior view and vertebral levels of the organs whose 3D images were obtained through segmentation are displayed.

Renal agenesis occurs as a result of the failure of the ureteric bud to fuse with the metanephric blastema during embryogenesis (Fig.1). Unilateral renal agenesis is seen in one in 1000-2000 live births, while bilateral renal agenesis is seen in one in 3000 to 4000 pregnancies.^11^ Studies have shown that there are symptoms such as hematuria and proteinuria in cases.^12^ In follow-up studies of patients with renal agenesis conducted in the Turkish population, has reported that renal agenesis is more common in men (68%-32%) 13 and is left-sided (64%).^14^ In another study, renal agenesis was observed in 9% of all CAKUT cases and renal hypoplasia in 3.8%.^15^ Studies on the anatomical disposition of these anomalies of the kidney tissue and how renal functions are affected by this condition are quite limited. Studies on the anatomical effects of congenital anomalies in the kidney tissue and how they affect kidney function in the Turkish population are limited. Our hypothesis was that anomalies in the development of the renal parenchymal tissue create anatomical differences in the structures associated with the kidneys. The aim of our study is to evaluate the anatomy of the retroperitoneal organs adjacent to the renal fossa in patients with UCRA and RH, to identify compensatory anatomic changes in the visceral tissues and to discuss their effects on renal functions.

## METHODOLOGY

Our study was conducted retrospectively with 18 renal agenesis, 30 renal hypoplasia group who were under the follow-up of İstanbul Dr. Lütfi Kırdar City Hospital Nephrology Clinic. The control group was selected from individuals who had CT scans for any reason and were confirmed to have no abdominal pathology, in accordance with age and gender distribution. The hospital’s OCTOMED database was used for all data. Morphometric measurements were obtained from contrast-enhanced computed tomography (CT) images by our expert instructors. All morphometric measurements were measured by two radiologists and final results were recorded by consensus.

### Ethical Approval:

The study was conducted with the permission of Health Science University Hamidiye Scientific Research Ethics Committee (Date: 13.10.2023, Decision: 18/17) and Istanbul Provincial Health Directorate (Date: 28.11.2023, Decision: 2023/19). Since the study was planned as a retrospective archive review, patient consent was not applied.

### Inclusion & Exclusion Criteria:

Individuals over 18 years of age who were diagnosed with unilateral renal agenesis, who did not have ectopic renal tissue, who did not undergo total nephrectomy, were included in the renal agenesis group. Individuals with a history of kidney transplantation, those who had undergone major abdominal surgery that could affect organs or vascular structures in the retroperitoneal space, those with artifactual CT images, those with scar tissue observed in radiological examination were excluded from the study.

### CT Features:

Computed tomography scan was performed using 128 multi-detector tomography device (Ingenuity 128 Slice, Philips Medical Systems, China). CT examination with intravenous contrast was performed after obtaining written consent. Patients were placed in the supine position. CT scans were obtained from the diaphragm to the symphysis pubis with 64 x 0.625 detector collimation, 1.016 pitch, 120 kV and 300 mAs parameters. Images were reconstructed with a 0.625 mm thick reconstruction interval. Images were obtained after a 20-35 second delay after the administration of 120 mL intravenous nonionic iodinated contrast. The entire abdomen was scanned with a single continuous breath hold within 10-12 seconds. The patient’s dose during the entire scan was measured as 1195.7 mGyxcm.

The obtained data were collected under three headings as demographic features, morphometric measurements and functional parameters.

### Morphometric Data:

After the CT data were obtained, 3D Multiplanar reconstruction images were created on the PACS system and morphometric measurements were performed. Anteroposterior (AP) and mediolateral (ML) diameters of healthy and affected kidneys were measured from axial sections, and craniocaudal (CC) diameters were measured from sagittal sections, and maximum values were recorded. Additionally, kidney volume (V) was obtained by segmentation using the 3D Slicer program. Cortex (Cx) thickness was obtained from the section with the maximum ML diameter in axial sections. The suprarenal gland was identified in the lateral neighborhood of the diaphragm crura, posterior to the IVC on the right, and anterosuperior to the kidney and behind the stomach on the left. Then, corpus, crus laterale and crus mediale measurements were made. Diameter measurements and locations of vascular structures [Vena cava inferior (VCI), Aorta abdominalis (Aa), A. renalis (A.r), V. renalis (Vr)] were examined in detail.

### Statistical Analysis:

SPSS v27 program was used for statistical analysis. Descriptive statistics were given for the sample. All data were evaluated with the Kolmogorov Smirnov test in terms of compliance with normal distribution. In normally distributed parametric data independent samples t-test was used to analyze data between two groups. ANOVA test was used to compare means between more than 2 groups, and post-hoc analysis was used for comparisons between groups. Chi-square test was applied to reveal the differences between categorical variables. Pearson correlation and linear regression analysis were used to determine the relationship between the data.

Statistical significance was accepted as p<0.05.

## RESULTS

The averages of morphometric measurements of the healthy side kidney, suprarenal gland, functional parameters and descriptive statistics between the groups are given in Table-I. The bifurcation angle of the aorta was the narrowest in the UCRA group and showed limited statistical significance against the RH individuals (UCRA: 38.74±12.85 degrees, RH: 46.49±12.32 degrees, Control: 41.70±9.54 degrees, p=0.071).

**Table-I:** Descriptive statistics and comparison of renal-vascular structures, suprarenal gland and functional parameters between groups.

	UCRA	RH	Control	P Value
N(%)	18 (24.65%)	30 (41.10%)	25 (34.25%)	
Age	35.67±15.90	40.07±12.62	39.32±11.77	0.520^b^
***Gender***				0.419^a^
Male	10	11	12	
Female	8	9	13	
***Affected Side***				0.328^a^
Right	11	15	-	
Left	7	15	-	
Variation	8/10	3/27	2/23	**0.003^a^**
***Morphometrics***				
A.ren Length (mm)	53.31±10.37	46.69±9.17	47.98±11.37	**0.007^b^**
A.ren Proximal Thickness (mm)	6.30±1.15	6.02±1.03	6.06±0.66	0.591
A.ren Distal Thickness (mm)	4.89±0.67	4.86±0.78	4.28±0.62	**0.005 b**
V.ren Length (mm)	60.63±23.65	55.41±25.21	81.82±11.48	**<0.01 b**
V.ren Proximal Thickness (mm)	8.08±1.96	6.74±2.06	6.89±2.27	0.091 **b**
A. Mesenterica Sup- AA Separation angle (Degree)	38.00±19.32	49.14±27.56	47.99±20.24	0.250 **b**
Kidney AP Diameter (mm)	64.31±9.69	61.10±9.24	62.71±7.08	0.459 **b**
Kidney CC Diameter (mm)	120.78±16.93	110.53±12.11	108.85±10.49	**0.009 b**
Kidney ML Diameter (mm)	64.29±11.01	60.81±6.72	48.44±6.82	**<0.001 b**
Kidney Volume (cm^3^)	225.05±80.65	207.87±53.94	167.57±43.52	**<0.001 b**
Total Kidney Volume (cm^3^)	255.05±80.65	262.45±64.25	331.13±77.90	**<0.001 b**
Cortex Thickness (mm)	17.51±3.62	17.38±1.92	15.12±1.49	**<0.001 b**
Gl. Suprarenalis Crus Lateralis (mm)	3.47±0.58	3.25±0.56	3.54±0.64	0.195 **b**
Gl. Suprarenalis Crus Medialis (mm)	3.29±0.58	3.46±0.72	3.69±0.69	0.159 **b**
Gl. Suprarenalis Corpus (mm)	5.66±0.98	5.57±1.38	6.64±1.04	**0.003 b**
***Biomarkers***				
Serum Urea (mg\dL)	32.46±14.17	32.49±18.05	25.38±6.75	0.133 **b**
Uric Acid (mg\dL)	5.92±1.33	4.87±1.36	4.14±1.35	**<0.001 b**
Sodium (mEq\L)	140.55±2.54	138.81±2.27	138.24±3.01	**0.017 b**
Potassium (mEq\L)	4.52±0.34	5.30±5.16	4.35±0.35	0.532 **b**
Creatinine (mg\dL)	1.07±0.56	5.07±1.00	0.76±0.15	0.182 **b**
eGFR (ml\min\1.73 m^2^)	91.64±30.00	94.92±24.93	109.49±15.37	**0.027 b**
Ph	6.19±0.42	6.23±0.40	6.03±0.42	0.195 **b**
Hemoglobin (g\dL)	13.55±1.81	12.54±1.86	13.24±1.80	0.147 **b**

Unilateral Congenital Renal Agenesis: UCRA, Renal Hypoplasia: RH, Arteria: A., Arteria abdominalis: AA, Vena: V., Anteroposterior: AP, Craniocaudal: CC, Mediolateral: ML, Glandula: Gl., Estimated Glomerular Filtration Rate: eGFR, P value ≤ 0.05: statistically significant, – value ≤ 0.01: highly statistically significant, Test applied =

a Chi-square,

b One-way ANOVA.(Tukey)

In the examinations made between the genders, except for the cortex thickness in terms of kidney measurements on the healthy side (AP, CC, ML length and volume), all other data (AP, CC, ML length and volume) showed that the kidney sizes in men were significantly higher (Table-II). It was observed that compensatory hypertrophy occurred from the control group individuals to the agenesis individuals and gave statistically significant results (p<0.001).

**Table-II:** In morphometric comparisons between genders, male kidney sizes were found to be significantly larger in all measurements except cortex thickness.

Healthy side	Sex	N	Mean	Std. Deviation	Std. Error Mean	p
AP Length(mm)	Male (M)	33	65.83	7.06	1.22	0.002^c^
	Female (F)	40	59.66	8.95	1.42	
CC Length(mm)	M	33	116.06	13.95	2.43	0.041^c^
	F	40	109.53	12.87	2.03	
ML Length(mm)	M	33	60.75	9.73	1.69	0.012 c
	F	40	54.70	10.14	1.60	
Cortex thickness(mm)	M	33	16.86	2.51	0.44	0.523 c
	F	40	16.47	2.62	0.41	
Kidney volume(cm^3^)	M	33	234.84	66.23	11.53	<0.001^c^
	F	40	181.67	57.58	9.10	

Anteroposterior: AP, Craniocaudal: CC, Mediolateral: ML, P value ≤ 0.05: statistically significant, – value ≤ 0.01: highly statistically significant, Test applied =

c Independent Sample T-Test.

VCI AP diameter was narrower in individuals with CAKUT. (The AP diameter average of VCI; 14.50±3.83 in 48 individuals with CAKUT, 17.10±3.80 in 25 individuals in the control group, p=0.007). Kidney volume in individuals with agenesis showed a positive correlation with GFR (r=0.504, p=0.017, Regression= 0.033). Regression equation: eGFR= 43.85 + (0.187)*V.

Cortex thickness stood out as the most important anatomical marker that was well correlated with functional-biochemical parameters of the kidney. Cortex thickness of the healthy side kidney showed positive correlation with eGFR (r= 0.633, p= 0.002) and negative correlation with creatinine level (r= -0.569, p=0.007) in individuals with congenital anomalies.

**Fig.2:**
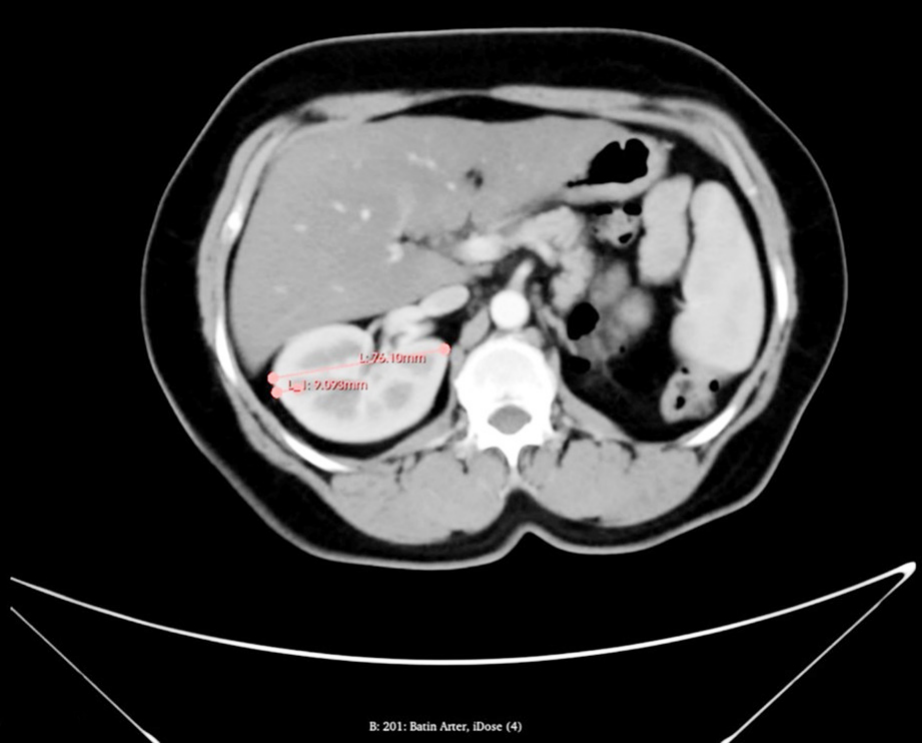
The cortex thickness/mediolateral diameter ratio was obtained from the section with the largest mediolateral diameter of the renal parenchyma in the axial plane and recorded.

In the whole sample, cortex thickness /ML length ratio (Cx/ML) showed negative correlation with urea (r= -0.255, p=0.029), uric acid (r= -0.236, p=0.044), creatinine (r= -0.295, p=0.011) and positive correlation with eGFR (r=0.346, p=0.003). The cut-off value of the Cx/ML ratio was calculated as 0.2991 (AUC: 0.706, 95% CI: 0.576, 0.836) with a sensitivity of 0.71 and a specificity of 0.69 (Fig.2).

**Fig.3:**
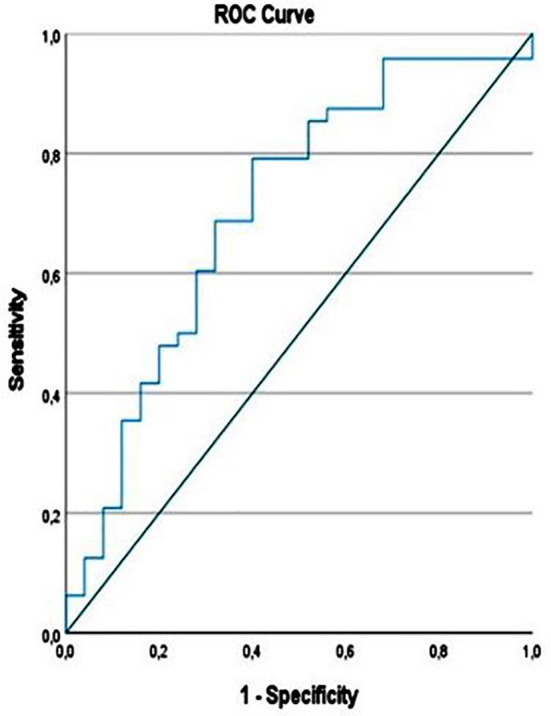
ROC Analysis of Cx/ML ratio.

## DISCUSSION

In this study, organs in the renal fossa and adjacent structures were examined. Measurements were performed with contrast-enhanced CT and individuals with unilateral congenital renal anomaly were selected as the patient group. The morphometric and functional effects that the absence (agenesis) or dysplasia of unilateral renal parenchymal tissue may have on the contralateral renal parenchyma were investigated. In addition, the associated vascular structures were evaluated. n the study, we found that individuals with kidney damage exhibited more frequent urinary system variations and a narrower angle at the aortic bifurcation. We also observed that the degree of compensatory hypertrophy increased with the size of the affected kidney. We evaluated the correlations of cortical thickness and the Cx/ML ratio with renal functional parameters. According to structure and function relationship studies, form helps function or function affects form. Various structural and functional scalings that will optimize energy use have been studied, proposed and validated.^16^

The thicker adrenal gland corpus in the control group compared to the CAKUT subgroups were significant. No studies on this topic were found in the literature. This difference observed in our study requires examining the possible compensatory effects of congenital anomalies in the kidney tissue on the adrenal gland.

In intrauterine development, the metanephric kidney tissue is important to turn 90 degrees and move cranially to the fossa renalis. Numerous studies have been conducted here to study the genetic basis of possible anomalies and to identify specific genes. According to Rosenblum et al., additional research and biomarkers are needed to properly follow CAKUT babies until adulthood.^17^ Westland et al. reported that 32% of patients with unilateral renal agenesis have different CAKUT pathologies and the most common is vesicoureteral reflux (24%). At the same time, 31% had extrarenal anomalies, 16% had hypertension, and 21% had microalbuminuria. GFR was <60 mL/min/1.73 m^2^ in 10% of the patients.^11^

### Anatomical markers of the kidney:

Nephron number, which is considered an important functional parameter of the kidney is determined during fetal life and does not increase after 34-36 weeks of gestation. According to Brenner’s hyperfiltration hypothesis, low nephron number is associated with compensatory hyperfiltration and hypertension in the remaining glomeruli, glomerular damage, and proteinuria, and may result in renal failure in the long term.^18^ Recent studies have attempted to estimate the number of nephrons in living human subjects, but more work is needed to obtain accurate and precise estimates of nephron numbers using noninvasive methods.^19^ Currently, there is no suitable method for determining the number of nephrons in living humans.^20^ In terms of anatomical markers, according to Denic et al., kidney volume is an inaccurate marker for estimating the number of nephrons.^21^

Kidney size is used in many clinical entities such as recurrent urinary tract infection, renovascular disease, vesicoureteral reflux, transplanted kidney follow-up, and donor evaluation. In cases such as chronic renal failure, treatment protocols are also tailored according to kidney size.^22^ Studies in donors reveal significantly higher renal blood flow in the remaining kidney compared to healthy controls, and MRI-based renal parenchymal volume has been shown to be a surrogate measure for renal function.^23^ There are many studies comparing kidney volume with functional parameters.^24^ In parallel with Funahashi, we showed that we can estimate GFR by obtaining total kidney volume in the regression analyses performed in the UCRA group in this study.

On average, 73% of the kidney volume is cortex and 27% is medulla. While cortex volume decreases until the age of 50, medullary volume increases and total kidney volume does not change.^25^ According to Noda et al., although parameters such as maximal kidney length show a positive correlation with eGFR, renal cortex thickness shows a stronger correlation than other anatomical markers.^26^ Here, we suggest that a Cx/ML diameter-length ratio taken from the axial plane can be used as an easily measurable and potential biomarker to reflect renal function. However, the AUC value of the Cx/ML ratio (0.706) indicates that this marker has moderate predictive power. Therefore, patient evaluation should be approached with this level of sensitivity. Prospective patient follow-up studies are needed for a more comprehensive and detailed evaluation of the Cx/ML ratio.

### Vascular Morphometry:

Although they were in the same age groups, it was observed that the VCI AP diameter was lower in individuals with CAKUT than in the healthy control group. This marker is evaluated by transabdominal US, especially in the emergency department, as an indicator of hemorrhagic or hypovolemic shock.^27^ We recommend that this patient group should be evaluated more carefully in terms of dehydration or that new percentile curves of VCI dimensions should be studied in this patient group.

Although current studies have revealed changes in parameters such as bifurcation angle, vertebral level and position of the ureter in cases such as horseshoe kidney,^28^ in our study, no significant anatomical effects were found in the surrounding tissues in the absence or smallness of kidney tissue. Song et al. demonstrated compensatory hypertrophy in the early postoperative period by showing a volume increase of 21.23% on the 3rd day and 24.17% on the 7th day in the healthy kidney in the uninephrectomy group.^29^ Compensatory hypertrophy can also occur in pathologies such as diabetes mellitus.^30^ In this study, we also demonstrated compensatory hypertrophy due to renal involvement, as expected.

### Limitations:

The single-center design and retrospective design of the study, the limited sample size, and the inability to assess concomitant systemic diseases due to the retrospective design are significant limitations. We also want to emphasize that the Cx/ML threshold should be validated in prospective studies. It should be noted that the narrower VCI AP diameter in the CAKUT group may have different pathological causes. The lack of data on the patients’ hydration levels during the measurements is also a significant limitation.

## CONCLUSION

In congenital renal anomalies such as UCRA and RH, anatomic effects on adjacent and related retroperitoneal organs have been found to be limited. The cortical thickness/mediolateral diameter (Cx/ML) ratio is considered a useful anatomic marker due to its correlations with urea, uric acid, creatinine, and estimated glomerular filtration rate (eGFR). However, this ratio requires validation in prospective studies to be considered a reliable indicator in clinical practice.
